# Prevalence and correlates of psychiatric morbidity, comorbid anxiety and depression among medical students in public and private tertiary institutions in a Nigerian state: a cross-sectional analytical study

**DOI:** 10.11604/pamj.2020.37.53.24994

**Published:** 2020-09-14

**Authors:** Joshua Falade, Adedayo Hakeem Oyebanji, Adefunke Olarinre Babatola, Olusola Olawumi Falade, Temitope Ojo Olumuyiwa

**Affiliations:** 1 Department of Mental Health, Afe Babalola University, Ado Ekiti, Ekiti State, Nigeria,; 2 Department of Pediatrics, Afe Babalola University, Ado Ekiti, Ekiti State, Nigeria,; 3 Department of Pediatrics, Ekiti State University, Ado Ekiti, Ekiti State, Nigeria,; 4 School of Nursing Osun state Hospitals´ Management Board Osogbo, Osogbo, Osun State Nigeria,; 5 Department of Community Health, Obafemi Awolowo University, Ile-Ife, Nigeria

**Keywords:** Comorbid anxiety and depression, psychiatric morbidity, medical students, private and public institutions, Nigeria

## Abstract

**Introduction:**

the study assessed the prevalence and factors associated with psychiatric morbidity (an array of psychological disorders), and comorbid anxiety and depression among medical students in Ekiti State, Nigeria.

**Methods:**

a cross-sectional study of medical students in two universities (one public and one private) in Ekiti state was conducted. A semi-structured questionnaire with adapted questions from the General Health Questionnaire and Hospital Anxiety and Depression Scale was used to obtain information on socio-demographic characteristics, psychiatric morbidity and comorbid anxiety and depression. Data was collected from April 2019 to August 2019. Data was analyzed using Statistical Package for Social Sciences (SPSS) version 21.

**Results:**

a total of 944 medical students participated in the survey. The overall prevalence of psychiatric morbidity and comorbid anxiety and depression among the respondents was 25.0% (CI = 22.1-27.8) and 14.3% (CI = 12.3-16.5) respectively. The factors independently associated with psychiatric morbidity included being a student of a private institution [adjusted odds ratio [AOR] =6.533, [95% confidence interval [C.I] =3.298-12.940], average academic performance [AOR =1.711, [95% C.I =1.173-2.496], below average academic performance [AOR =2.425, [95% C.I =1.313-4.478], and having a father or a mother with highest level of formal education below first degree [AOR =3.147, [95% C.I =1.579-6.272] and [AOR =2.053, [95% C.I =1.074-3.927] respectively. The factors independently associated with comorbid anxiety and depression were being a student receiving less than one dollar equivalent per day as allowance [AOR = 1.953, [95% C.I = 1.135-3.360] and being a student from the Igbo ethnic group [AOR = 0.533, [95% C.I = 0.333-0.853].

**Conclusion:**

the prevalence of psychiatry morbidity and comorbid anxiety and depression was high among medical students in Ekiti State, Nigeria. Periodic medical [mental health] screening for medical students may be appropriate to screen, detect and manage psychiatric comorbidities. This will help to ensure optimal mental health for this group of university students.

## Introduction

Medical education encompasses training to become a medical practitioner; or a specialist. It requires mastering large volume of information, a longer duration of training and more intensive method of assessment [1]. Many medical students struggle with their own capacity to meet the demands of the curriculum [2-4]. It is also imperative for them to be balanced psychologically to ensure success in the training and future practice of medicine [5]. Medical schools are known to be stressful environments for students and hence medical students have been believed to experience greater incidences of psychological disorders [6]. Depression and anxiety are among the common mental health problems among medical students and are associated with poor academic performance, disability and poor quality of life. A better understanding of the magnitude and correlates of depression and anxiety is essential for planning appropriate intervention for those population groups. Globally, studies have reported high prevalence of psychiatric morbidity anxiety and depression among medical students [7-9]. The global prevalence of depression amongst medical students was 28.0% [6]. In the developed countries Becker found that 37% of young adults between the ages of 15 to 24 years in the United States of America had a diagnosable mental disorder, and many of these individuals were college students [10].

In Nigeria a study in Ibadan indicated that 12% of pre-clinical students [10] and 21% of clinical students had psychiatric morbidity [10], while Makanjuola *et al*. reported that 14.7% of the medical students in Ilorin had psychiatric morbidity [11], Oshodi *et al*. reported 42.2% of medical students in Lagos to have psychiatric morbidity [12] while Oku *et al*. reported that 39.2% of medical student in Calabar had poor mental health [13]. Previous research has identified long hours of study, and the impositions of emotional burden [14], high workload [15] and considerable financial pressure as the principal stressors. Omigbodun *et al*. found that the prevalence of psychological distress was be higher among male students. Other socio-demographic factors associated with psychological morbidity include being in a transition year of study, reporting financial distress and not being a ‘Pentecostal Christian’, excessive school work, congested classrooms, strikes by faculty, lack of laboratory equipment, family problems, insecurity, and financial and health problems. They also noticed that medical and dental students had significantly higher GHQ scores than the physiotherapy and nursing students [16]. There is paucity of data on psychiatric morbidity and co-morbid anxiety and depression among students in medical schools in Nigeria especially private medical schools. The study therefore assessed the prevalence and factors associated with psychiatric morbidity, and comorbid anxiety and depression among medical students in Ekiti State, Nigeria.

## Methods

**Study settings, participants and design:** this was a cross sectional study which involved public and private medical schools in Ekiti State southwestern, Nigeria. There are two medical schools in the state; Ekiti State University a public non-residential institution owned by the state government and Afe Babalola University, a private residential university owned by Chief Afe Babalola. Both universities are located in the State Capital (Ado Ekiti) of Ekiti state. The 2 medical schools in Ekiti State were purposively selected and all 965 students in second year through sixth year in both schools were invited to participate in the study. All second to sixth year-medical students in both universities who gave informed consent were enrolled. Having retrieved the students´ class lists, each participant was approached before the lecture hours or the hospital´s Ground Round for the students in clinical postings to explain the purpose of the study, give assurance of confidentiality and to explain the benefits of the study. Thereafter, the informed consent form and the self-administered questionnaire were given to each participant. Each questionnaire was checked by the researchers and trained research assistants for adequate completion. A few students were asked to fill identified blank spaces in the questionnaire before final submission. The study spanned from April 2019 to August 2019.

### Study instruments

**The socio-demographic schedule/proforma:** this section contained information on the socio-demographic profiles of respondents. It contained the following items: age, gender, religion, marital status, a self-rated academic performance grouped as follows: -below average [1-4], average (5 and 6) and above average (7-10). The schedule also includes sources of finance, marital status of the parent, family type and parents´ level of education and occupation.

**The general health questionnaire (GHQ):** the David Goldberg designed GHQ: a self-administered screening instrument for detecting non-psychotic psychiatric disorders, focusing on the inability to carry out normal function. The 12-item General Health Questionnaire (GHQ-12) has been extensively used as a short screening instrument, producing results that are comparable to longer versions of the GHQ. In a study, the validity of the GHQ-12 was determined against the Composite International Diagnostic Interview (CIDI). It has a sensitivity of 68% and specificity of 70% [17, 18]. The 12-item version of the instrument was used in this study. The GHQ-12 is scored using the binary method (0-0-1-1). Each item has four possible responses, typically being ‘not at all’, ‘no more than usual’, ‘rather more than usual’ and ‘much more than usual’. Therefore a score of 3 and above will be used as an indication of psychiatric morbidity [19]. In a study by Gureje, the alpha coefficient of the GHQ-12 was 0.82 [18].

**Hospital anxiety and depression scale (HADS):** the HADS is a self-report instrument efficiently used to assess depression and anxiety [17]. In a Nigerian study, the sensitivity for the anxiety sub-scale ranged from 85.0% to 92.9%, while sensitivity for the depression sub-scale ranged from 89.5% to 92.1% [18]. The HADS is considered to be unaffected by coexisting general medical conditions [19], unlike GHQ items where symptoms may refer to physical cause like insomnia and weight loss. For GHQ 12, using 3 as cut-off point, ≥3 is “possible psychiatric morbidity [i.e. ‘GHQ 12 cases’] and <3 as no morbidity [i.e. ‘GHQ 12 non-cases’]”. The Hospital Anxiety and Depression Scale will be scored accordingly 0-7 = normal, 8-10 = borderline abnormal, 11-21 = abnormal. Respondents with borderline abnormal and abnormal cases were considered as having anxiety or depression.

**Ethical consideration:** ethical approval was obtained from the Research Ethics Committee of the Ekiti State University Teaching Hospital Ethics and Research Committee. [Protocol Number-EKSUTH/A67/2019/01/005] and Afe Babalola University Participation was voluntary and informed consent was obtained from the study participants.

**Data Analysis:** the Statistical Package for Social Sciences (SPSS version 21) was used for Data analysis. The socio-demographic details of respondents were reported using descriptive statistics. Categorical variables were summarized using frequencies and percentages while continuous variables were summarized using means and standard deviation. Pearson’s chi-square test was used to determine the association between comorbid anxiety and depression, psychiatric morbidity and socio-demographic variables. The independent variables entered into the binary logistic regression model were those that were significant at 5% (p<0.05) on bivariate analysis and other factors that have been found to be significant predictors from literature (e.g. age and sex) [20-22]. Confidence interval was set at 95% and all tests were two-tailed. Statistical significance was considered at a p-value less than 0.05.

## Results

There were 965 eligible medical students in both institutions, out of which 944 (97.8%) of them participated in the study. Five hundred and twelve (54.2%) of the respondents were aged 10-20years while 432 (45.8%) were aged 21 years and above. The age range of the medical students was between 16 and 32 years, overall mean was 21 ±3.0 years, with mean age of 19.8±1.8years and 25.25±2.7 years in the Private and Public institutions respectively. Most of the respondents (78.4%) attend the private tertiary institution. Female, Christian, and single were 68.0%, 81% and 95.6% respectively. The majority, (94.0%) are financed by their parents, over half (51.2%) of the respondents were from Yoruba ethnicity, 449 (47.6%) were from the Igbo ethnicity and 12 (1.2%) were Hausas. Five hundred and forty-six (57.8%) were in pre-clinical stage (second and third year medical students) of the medical training while 398 (42.2%) were in the clinical years. Five hundred and twenty-five (55.6%) described themselves as academically average, 347 (36.8%) above average and 72 (7.6%) below average. About one-tenth (100) spend equivalent of one dollar or less per day while the rest 884 (89.4%) spend more than one dollar daily. Six hundred and forty-five (68.3%) were satisfied with monthly allowance. A significant proportion (91.8%) were from monogamous background, With regard to the respondents’ parents highest level of formal education; 853 (90.4%) of their mothers had highest level of education being tertiary education while the rest 91(9.6%) as no tertiary education (none, primary and secondary education). Eight hundred and seventy four (92.6%) of their fathers had highest level of education being tertiary education while the rest 70 (7.4%) had no tertiary education.

**Prevalence of psychiatric morbidity and comorbid anxiety and depression of the respondents:** the overall prevalence of psychiatric morbidity and comorbid anxiety and depression among the respondents was 25.0% (21.1-27.8) and 14.3% (12.1-16.5) respectively. The prevalence of psychiatric morbidity among respondents from the public institution was 11.8% (7.5-16.6) while it was 28.6% (25.3-32.2) among respondents from the private institution. The prevalence of comorbid anxiety and depression among respondents from public and private institution was 27% (20.8-33.2) and 10.8% (8.6-13.2) respectively.

**Association of comorbid anxiety and depression with socio-demographic characteristics of the respondents:** Table 1shows comparison between 135 subjects with anxiety and depression and 809 without. More participants aged 21years and above (59.3%) had comorbid anxiety and depression compared to those below 21years, this difference was statistically significant [p =0.001]. A higher proportion of medical students in public university had comorbid anxiety and depression compared to those in the private tertiary institution, this was statistically significant [p< 0.001]. A significant number who were Muslims had comorbid anxiety and depression compared to others of other religion, this was also statistically significant [p =0.004]. Respondents who were of the Igbo ethnic group were less frequently affected by anxiety and depression compared to other ethnicities, this difference was statistically significant [p <0.001]. More respondents who described themselves as being below average academically were more likely to have had anxiety and depression compared to others, this difference was statistically significant [P<0.001]. Respondent that spent one dollar or less per day had significant comorbid anxiety and depression than those with more than one dollar daily [<0.001].

**Table 1:** association of comorbid anxiety and depression with socio-demographic characteristics of the respondents

Variable	Anxiety and depressionabsentn [%]	Anxiety and depression presentn [%]	P-value
**Age group (years)**			
10-20	457 [56.5]	55 [40.7]	**0.001**
21 and above	352 [43.5]	80 [59.3]	
**Institution type**			
Private	660 [81.6]	80 [78.4]	**<0.001**
Public	149 [18.4]	55 [21.6]	
**Gender**			
Male	256 [31.6]	46 [34.1]	0.575
Female	553 [68.4]	89 [65.9]	
**Marital status**			
Single	775 [95.8]	127 [94.1]	0.369
Married	34 [4.2]	8 [5.9]	
**Ethnicity**			
Yoruba	388 [48.0]	102 [71.8]	**<0.001**
Igbo	413 [51.0]	36 [25.4]	
Hausa	8 [1.0]	4 [2.8]	
**Religion**			
Christianity	670 [82.8]	98 [72.6]	**0.004**
Islam	131 [16.2]	37 [27.4]	
Traditional	8 [1.0]	0 [0.0]	
**Stage**			
Pre-clinical	476 58.8]	70 [51.9]	0.128
Clinical	333 [41.2]	65 [48.1]	
**Academic performance**			
Below average	58 [7.2]	30 [19.9]	**<0.001**
Average	461 [57.0]	64 [42.4]	
Above average	290 [35.8]	57 [37.8]	
**Source of finance**			
Parents	762 [94.2]	125 [92.6]	0.602
Other family members	3 [0.4]	1 [0.7]	
Self	18 [2.2]	3 [2.2]	
Spouse	26 [3.2	6 [4.4]	
**Average amount spent**			
≤ one dollar per day	69 [8.5]	31 [23.0]	**<0.001**
> one dollar per day	740 [91.5]	104 [77.0]	
**Satisfied with monthly allowance**			
Yes	554 [68.5]	91 [67.4]	0.804
No	255 [31.5]	44 [32.6]	
**Parents marital status**			
Single parent	32 [4.0]	15 [6.5]	0.165
Married	713 [88.1]	206 [88.8]	
Divorce/separated	26 [3.2]	4 [1.7]	
Widow/widower	38 [4.7]	7 [3.0]	
**Family type**			
Monogamy	747 [92.3]	120 [88.9]	0.176
Polygamy	62 [7.7]	15 [11.1]	
**Fathers educational level**			
No tertiary education	60 [7.4]	8 [5.9]	0.531
Tertiary education	747 [92.6]	127 [94.1]	
**Mothers educational level**			
No tertiary education	81 [89.0]	10 [11.0]	0.342
Tertiary education	728 [85.3]	125 [14.7]	

**Association of psychiatric morbidity with socio demographic characteristics of the respondents:** Table 2 shows comparison between 212 subjects with psychiatric morbidity and 528 without. A significant proportion who attends private tertiary institutions (28.6%) had psychiatric morbidity compared to respondents in a public institution, difference was statistically significant [p <0.001]. One hundred and seventy-five (27.3%) female respondents had psychiatric morbidity compared with 61 (20.2%) male respondents, this was statistically significant [p =0.020]. Traditional worshippers (50.0%) had statistically significant psychiatric morbidity compared to other religions [p <0.001]. Hausa respondents were more likely to be affected by psychiatric morbidity, this difference was statistically significant [p =0.013]. Additionally, more respondents who described themselves as being below average in academic performance had significant psychiatric morbidity than those who described themselves as being average or above average academically. This was statistically significant [p <0.001]. One hundred and four (34.8%) respondents, not satisfied with their monthly allowance had psychiatric morbidity compared to one hundred and thirty-two (20.5%) who were satisfied, this was statistically significant [p value<0.001]. Respondents with parents (father or mother) with highest level of formal education as tertiary education were less frequently affected by psychiatric morbidity compared to those with parents with no tertiary education, the difference was statistically significant, [p value<0.001 and p =0.001 respectively]. Respondents who were not satisfied with their monthly allowance were more likely to be affected by psychiatry morbidity, the difference was also statistically significant [p<0.001].

**Table 2:** association of psychiatric morbidity with socio-demographic characteristics of the respondents

Variable	No Psychiatric morbidityn [%]	Psychiatric morbidity presentn [%]	P value
**Institution type**			
Private	528 [74.6]	212 [89.8]	<0.001
Public	180 [25.4]	24 [10.2]	
**Gender**			
Male	241[34.0]	61 [25.9]	0.020
Female	467[66.0]	175[74.2]	
**Marital status**			
Single	674 [95.2]	228 [96.6]	0.362
Married	34 [4.8]	8 [3.4]	
**Ethnicity**			
Yoruba	387 [54.2]	102 [43.2]	0.013
Igbo	319 [44.7]	130 [55.1]	
Hausa	8 [1.1]	4 [1.7]	
**Religion**			
Christianity	554 [78.2]	214 [90.7]	<0.001
Islam	150 [21.2]	18 [7.6]	
Traditional	4 [0.6]	4 [1.7]	
**Stage**			
Preclinical	419 [59.3]	127 [53.8]	0.136
Clinical	287 [40.7]	109 [46.2]	
**Academic performance**			
Below average	42 [5.9]	30 [12.7]	<0.001
Average	373 [52.7]	152 [64.4]	
Above average	293 [41.4]	54 [22.9]	
**Average monthly allowance**			
≤ One dollar per day	82 [11.6]	18 [7.6]	0.087
>One dollar per day	626 [88.4]	218 [92.4]	
**Satisfaction with allowance**			
Yes	513 [72.5]	132 [55.9]	<0.001
No	195 [27.5]	104 [44.1]	
**Parents marital status**			
Single parent	37 [5.2]	10 [4.2]	0.794
Married	616 [87.0]	206 [87.3]	
Divorce/separated/widowed	55 [4.2]	20 [0.0]	
**Fathers educational level**			
No tertiary education	37 [5.2]	33 [14.0]	<0.001
Tertiary education	671 [94.7]	203 [86.0]	
**mothers educational level**			
No tertiary education	55 [7.8]	36 [15.3]	0.001
Tertiary education	653 [95.2]	200 [84.7]	

**Socio-demographic and clinical variables independently associated with psychiatric morbidity by logistic regression analysis:** the factors independently associated with psychiatric morbidity included academic performance below average and average which was about 2.4 folds and 1.7folds respectively increased odd of psychiatric morbidity[Adjusted odds ratio [AOR] =2.425, [95% confidence interval [CI] =1.313-4.478] and [AOR] =1.711, [95% C.I = 1.173-2.496] respectively. Besides, being a student at a private institution has about 6.5 odd increase of psychiatric morbidity compared with their counterparts in the public institution [AOR = 6.533, [95% C.I = 3.298-12.940]. Students whose fathers didn´t have tertiary education [AOR = 3.147, [95% C.I = 1.579-6.272], students whose mothers didn´t have tertiary education [AOR = 2.053, [95% C.I = 1.074-3.927] and students who were not satisfied with their monthly allowance [AOR =2.2135, [95% C.I = 1.495-3.049] were more likely to have psychiatric morbidity. However, Muslims were less likely to have psychiatric morbidity [AOR = 0.348, [95% C.I = 0.194-0.626] (Table 3).

**Table 3:** the socio-demographic and clinical variables independently associated with psychiatric morbidity by logistic regression analysis

Variable	P-value	Odd ratio (95% Confidence Interval)
		
**Tribe**		
Yoruba [ref]		
Hausa	0.480	1.646 [0.413-6.561]
Igbo	0.081	0.725 [0.505-1.040]
**Religion**		
Christianity [ref]		
Islam	<0.001	0.348 [0.194-0.626]
Traditional	0.315	2.163 [0.480-9.744]
**Marital status**		
Married[ref]		
Single	0.565	1.324 [0.509-3.442]
**Institution**		
Public[ref]		
Private	<0.001	6.533 [3.298-12.940]
**Academic performance**		
Above average [ref]		
Below average	0.005	2.425 [1.313-4.478]
Average	0.005	1.711 [1.173-2.496]
**Average Satisfaction**		
Yes [ref]		
No	<0.001	2.135 [1.495-3.049]
**Fathers level of education**		
Tertiary education [ref]		
No tertiary education	0.001	3.147 [1.579-6.272]
**Mothers level of education**		
Tertiary education [ref]		
No tertiary education	0.030	2.053 [1.074-3.927]
**Gender**		
Female [ref]		
Male	0.241	0.802 [0.555-1.159]
**Age (years)**		
10-20 [ref]		
21 and above	0.104	0.733 [0.505-1.066]

**Association between comorbid anxiety and depression and other variables by logistic regression analysis:** medical students who spent equivalent of less than one dollar per day were approximately 2 times more likely to report symptoms of comorbid anxiety and depression [AOR] =1.953, [95% C.I = 1.135-3.360] while respondents of Igbo ethnic groups were less likely to report symptoms of anxiety and depression [AOR] = 0.533, [95% C.I = 0.333-0.853] (Table 4).

**Table 4:** association between comorbid anxiety and depression and other variables using logistic regression

Variable	P-value	Odd ratio (95% Confidence Interval)
**Age group (in years)**		
10-20 [ref]		
21 and above	0.594	1.138 [0.708-1.830]
**Gender**		
Male [ref]		
Female	0.745	1.069 [0.714-1.601]
**Tribe**		
Yoruba[ref]		
Hausa	0.109	2.835 [0.792-10.145]
Igbo	**0.009**	0.533 [0.333-0.853]
**Religion**		
Christianity [ref]		
Islam	0.395	1.226 [0.766-1.963]
Traditional	0.999	0.000 [0.000-.]
**Marital status**		
Married [ref]		
Single	0.805	1.115 [0.468-2.661]
**Institution**		
Private [ref]		
Public	0.121	1.588 [0.885-2.849]
**Monthly allowance**		
More than a dollar per day [ref]		
Less than one dollar per day	**0.016**	1.953 [1.135-3.360]

## Discussion

The mean age of the medical students in Ekiti State was 20.9 years, those in the public institution were 5.45 years older than those in the private institution. Most of the respondents were in pre-clinical years and less than 20 years. The mean age of the medical students in Ekiti State is lower than 26.9 years and 23.91 years reported by previous studies in the public institutions [23,24]. The mean age of medical students in the public institution in this study is however similar to previous reports. There is a dearth of study among students in private institutions. More females were found in this study compared to earlier reports for public schools [23,25,26]. This may be a new trend in gender distribution or due to the recent national campaign and advocacy for girl child education. Most of the respondents were single probably due to the age range, high parental literacy level and tradition i.e the Yorubas prefers females to complete first degrees before marriage to honour the parents. The socio-economic instability of the nation and parental viewpoint may be responsible for dissatisfaction with monthly allowances or dissatisfaction may be from comparison with fellow rich medical students. Poor remuneration from parents may be responsible for spending less than one dollar per day. Most of the respondents were average students. Since 50% score is required for tests and examinations, most students will therefore describe themselves as average. This observation is similar to a previous report in southwestern Nigeria [27].

The prevalence of psychiatric morbidity among the medical students in this study was 25%, a potential for undesirable consequences now and in future for the students [28]. A prevalence of 11.8% in this study among medical students in the public institution is comparable with 12.0% reported among preclinical students but lower than 21.0% and 45% among clinical students in two public medical institutions, in Ibadan and Lagos, Nigeria [14-16]. These two towns are cosmopolitan cities when compared with this study location. Other public medical institutions in Nigeria reported high prevalences [29, 30]. The prevalence of psychiatric morbidity among medical students in the private institution was 28.6% and this is higher than the 11.8% reported in the public institution. It is lower than 46.2% reported in a private medical institution in Malaysia [31]. There is paucity of studies among medical students in private institutions in Nigeria, this is probably because many private institutions may think that these reports can tarnish the image of their university.

Factors such as academic performance, religion, satisfaction with monthly allowance and institutional type were individual predictors of psychiatric morbidity identified in this study. Medical education requires mastering of tremendous amount of information, spending more years in training, scoring higher grades with ability to recall information from previous courses [32]. Inability to handle necessary information may result in struggles with the medical curriculum and psychiatric morbidity [16]. Respondents who described themselves as average and below-average were more likely to report symptoms of psychiatric morbidity [33]. Religion is an important concept in mental health commonly used as coping strategy [27]. In this study Muslims were less likely to report symptoms of psychiatric morbidity, probably due to an underlining moral necessity for protection and care of the vulnerable individuals inherent within it [34]. Parental education influences children´s manner, actions and motivation. In this study, respondents whose parents highest level of formal education were below first degree were more likely to be affected by psychiatric morbidity like in earlier studies [35].The level of education may affect the rate understanding, relationship and financial capacity. Parents who do not have tertiary education are less likely to understand what it takes to be a doctor even emotionally.

In this study respondents not satisfied with their monthly allowances were more likely to report symptoms of psychiatric morbidity. This non-satisfaction may be relative to the student’s lifestyle, ostentatious background, future expectations and other psychosocial factors. Financial satisfaction may be improved with holiday jobs, scholarships, management skills training and subsidized tuition. Respondents in the private institution were more likely to report symptoms of psychiatric morbidity in this study. Medical students are relatively younger in private institution than in public university. College life for teenagers may impose major challenges to studying and socializing and failure to manage these challenges may lead to poor mental health with repercussions [36]. Private universities have stricter rules, restriction of movement and freedom and stiffer punishments for disobedience. Private universities in Nigeria are very expensive with a myriad of fees. The whole process of raising funds for school may be stressful for both parents and students. This may be a source of psychiatric morbidity.

In this study the prevalence of comorbid anxiety and depression among the respondents was 14.3%, it was 27.0% in public institution against 10.8% in private institution. Medical students have been found to experience higher levels of depression and anxiety compared to other students or the general population [4]. In Pakistan, a study conducted among private medical students found prevalence of anxiety and depression using the Aga Khan University Anxiety and Depression Scale [AKUADS] of 4^th^ year, 3^rd^ year, 2^nd^ year and 1^st^ year to be 49%, 47%, 73%, and 66% respectively [37]. A higher prevalence of anxiety and depression [43.89%] was found amongst students of Nishtar Medical College, Multan with prevalence of anxiety and depression specifically found to be 45.86%, 52.58%, 47.14%, 28.75% and 45.10% among students of first, second, third, fourth and final years respectively [38]. The consensus from all the previous studies, responsible for high prevalence of anxiety and depression among private medical students were competitiveness and demand for high grades thus students are pushed to work harder. This may be to justify their high tuition and to attract future potential students. The majority of medical students have financial indebtedness to school and financiers. There are also poor leisure activities and exercise, especially for fresher and final year students. These may be due to pressure from parents as a result of higher tuition [39]. Additionally, high parental expectations also cause undue stress [40].

In this study, more respondents from public institution reported symptoms of comorbid anxiety and depression. Tuition fees are lower in public institutions than in private institutions, however incessant industrial actions relating to staff salaries and infrastructure issues prolong students’ stay in school. The vast majority of students in public universities often come from low socio-economic backgrounds, may be exposed to chronic poverty with large families. Respondents who spend less than one dollar equivalent per day in this study were more likely to be affected by comorbid anxiety and depression. Medical education is financially demanding, non-affordability of basic needs from living below the WHO poverty line is stressful and a template for comorbid anxiety and depression. Respondents from the Igbo tribe in this study were less likely to have comorbid anxiety and depression than the Yoruba tribe. The Igbos are predominantly businessmen and women who may have the financial strength to make their wards comfortable in the school.

## Conclusion

Comorbid depression and anxiety, and psychiatric morbidity are common among Nigeria medical students, both in private and public institutions. In this study, a variety of predictors as well as protective factors were identified. An effective model for prevention and early detection of depression, anxiety and psychiatric morbidity among medical students need to be developed. Policymakers, school administrators, government, parents, academic governing body and students should work together to reduce these high prevalence rates to improve the mental health of our future doctors.

### What is known about this topic

- Prevalence of psychiatric morbidity anxiety and depression is high among medical students globally;
- Long hours of study, high workload, the impositions of emotional burden and considerable financial pressure are principal stressors.

### What this study adds

- The prevalence of comorbid anxiety and depression is higher among students in public medical school than private medical school;
- The factors independently associated with psychiatric morbidity included being a student with self-reported description of being below and average academically, and being a student in a private institution;
- The factor independently associated with comorbid anxiety and depression was being a student receiving less than one dollar equivalent per day.
